# Pipette Show: An Open Source Web Application to Support Pipetting into Microplates

**DOI:** 10.1021/acssynbio.1c00494

**Published:** 2022-01-13

**Authors:** Johannes Falk, Marc Mendler, Johannes Kabisch

**Affiliations:** † Department of Life Sciences & Chemistry, Jacobs University Bremen, Campus Ring 1, 28759 Bremen, Germany; ‡ Institut für Physik Kondensierter Materie, Technische Universität Darmstadt, Hochschulstr. 6, 64289 Darmstadt, Germany; § Department of Biotechnology and Food Science, Trondheim − Gløshaugen NTNU, Sem Sælandsvei 6-8, Kjemiblokk 3, 7034 Trondheim, Norway; ∥ Computer-Aided Synthetic Biology, TU Darmstadt, 64289 Darmstadt, Germany

**Keywords:** laboratory workflow, microplates, digital lab, deskilling, reproducibility

## Abstract

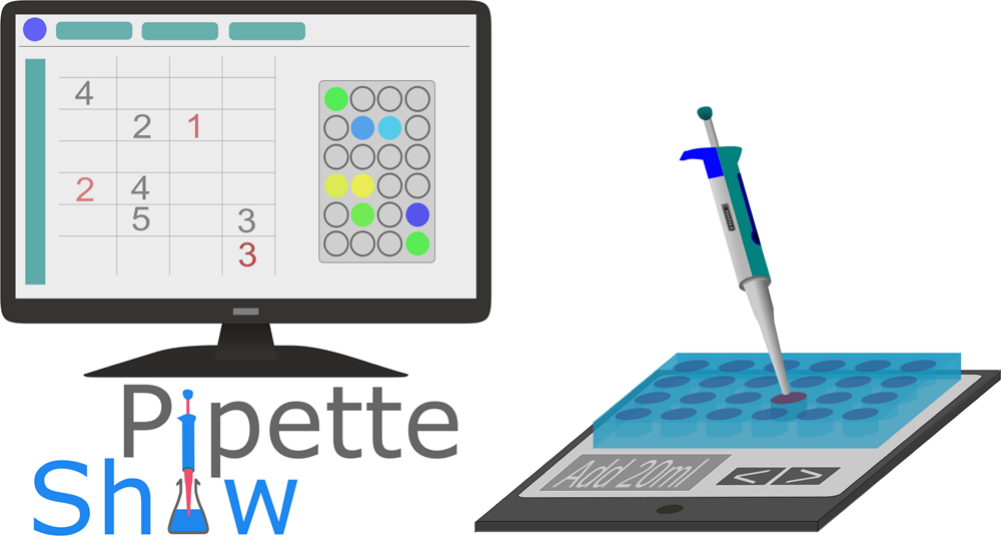

Despite increasing automation, manual pipetting remains a daily important task in life science laboratories. However, the creation of an efficient work plan is often time-consuming, and its completion is error-prone. Here, we present Pipette Show, a free Vue.js based application that optimizes the generation of an efficient work plan for pipetting into microplates and supports its reliable execution by visual guidance. The basis forms a graphical web interface with a module for building workflows as well as a module displaying the information for each pipetting step by illuminating wells of microplates placed on a tablet.

## 1 Introduction

Experiments in biology and medicine are required to be reproducible, as well as smooth and fast in respect to their execution.^1^ To enable the repetition of experiments and eliminate variations in the experimental procedure, meticulous documentation of the steps to be carried out is necessary.^2,3^

For high reproducibility, various procedures, such as a detailed laboratory notebook and the preparation of reports, are used. It is also the aim of various open and commercial software projects to support researchers in the precise documentation and the repetition of an already documented experiment. The Aquarium project, for example, offers a special language to describe replicable laboratory instructions and generates step-by-step guides to ensure that protocols will be run the same way every time.^4^

However, research studies have shown how monotonous work like pipetting into 96-well plates can quickly cause fatigue and thus foster errors like the confusion of wells, ultimately leading to nonreproducible and false results.^5^ Consequentially, especially the drop in the price of robotic hardware has led to increasing automation of laboratory work steps.^6,7^ Since then, tedious and repetitive work like pipetting tasks are often no longer performed by human hands and can thus be carried out more reliably and faster. However, especially for the most commonly used 96-well format and small test series or proof of concepts, manual pipetting is still indispensable and often faster than establishing a liquid-handler workflow. Additionally, for many researchers in developing countries manual pipetting is the only method available or the supply of robotic tips are scarce.

Here, we present Pipette Show, a software tool that addresses this point. Pipette Show makes it possible to define pipetting steps and helps to execute them error-free and thus reproducibly. At the same time, Pipette Show supports a faster execution of work. The software is based on two modules: the *Build* module is optimally run on a computer and allows to plan the pipetting steps required for an experiment. The finished instructions can be loaded into the *Show* module, which is designed to be run on a tablet. After mounting a well plate onto the tablet, Pipette Show guides each pipetting step by back-lighting the relevant wells as well as informing the user of the liquid and volume to be transferred.

There are already commercial projects that pursue similar strategies. With My Pipette Creator App and PipettePilot, the companies Gilson and Thermo Scientific offer applications that connect to their electronic pipettes and subsequently guide the user through the protocol, e.g., by back-lighting.^8,9^ Similarly, BioSystemika developed the pipetting aid PlatR, an android app that is sold together with a tablet and accessories.^10^ All these products provide functionalities to create protocols and share them between users. Additionally, their visual guidance helps to pipet faster and more reliably—according to BioSystemika, using PlatR can increase the throughput by up to 50%. However, since all these applications are proprietary, they are not only costly and bound to specific devices, but there is also no means to adjust the functionality to specific needs. With iPipet there is also a free and open source web application available.^911^ Users can upload offline generated CSV-files with pipetting instructions. After aligning a well plate on a tablet computer, iPipet visually supports pipetting—similar to Pipette Show—by illuminating active wells. Table 1 compares all these solutions with our tool.

In contrast to the commercial products, being a Vue.js application, Pipette Show is device independent and can be fully run in many modern browsers. Furthermore, Pipette Show is open source and expandable in all areas, e.g., by developing new instruction generating plugins, by adding other well-plate formats or by providing functionalities to import data from different sources. At the moment, for example, Pipette Show offers a rudimentary import for files generated by a dispensing module of DIVA (https://public-diva.jbei.org/) or within the Opentrons Protocol Designer (https://designer.opentrons.com/). In contrast to the proprietary solutions, Pipette Show can be used with any pipette and is not bound to vendor-specific hardware.

**Table 1:** Comparison of Different Available Pipetting Tools

name/company	open source	protocol creator	visual guidance	public protocols	free	comments
My Pipette Creator App/Thermo Fisher Scientific	No	Yes	No	Yes	No	Requires E1-ClipTip Pipettes
PipettePilot/Gilson	No	Yes	Yes	No	No	Requires PIPETMAN M Pipettes
PlatR/BioSystemika	No	Yes	Yes	No	No	–
iPipet	Yes	No	Yes	Yes	Yes	–
Pipette Show	Yes	Yes	Yes	No	Yes	Text based protocols for easy sharing

## 2 *Build* Module

**Figure 1:**
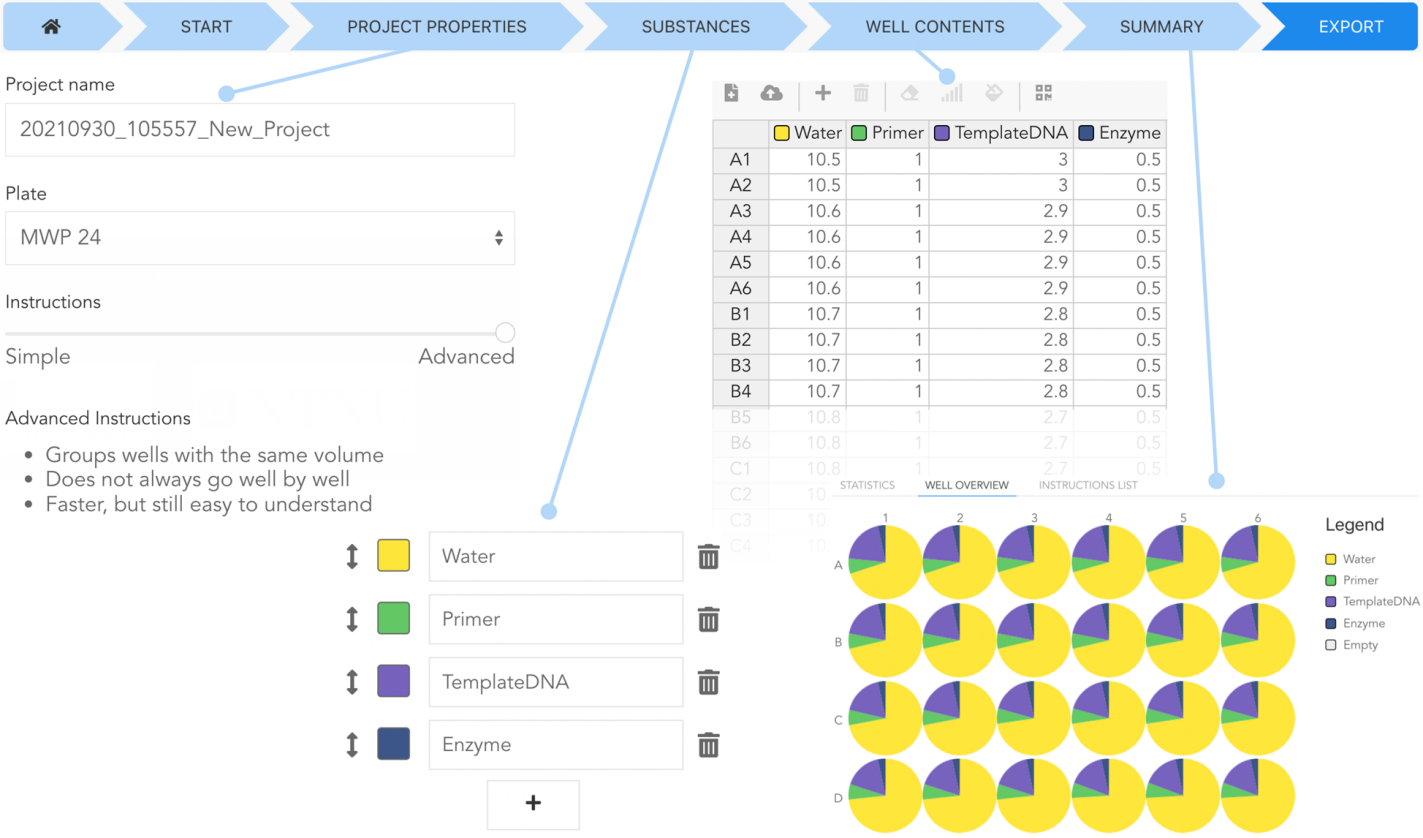
Selected interfaces of the *Build* module. A progress bar on the top indicates the current task. *Project Properties*: A name is autogenerated and can be edited, a choice of plate formats is available, and different modes for pipetting can be chosen. *Substances*: The user can add, rename, or rearrange the substances to be pipetted, and colors are autogenerated but can be edited by color picking or color codes. *Well Contents*: A spreadsheet page allows the amount of the substances to be modified. Additional substances can be added, files up- or downloaded, backfill and gradient options applied. *Summary*: Overviews of the designed experiment. Not shown: *Start*: The user can import various file formats or generate new instructions; *Export*: The user can download the instructions or transfer them via QR-code, link, or Google Drive to the *Show* module.

Within the *Build* module (Figure 1), new experiments are designed. Besides starting from scratch, it is also possible to open an existing Pipette Show (.PIP) file, or to import data from various source formats. Currently, Pipette Show can import experiments from .CSV and .XLS(X) files as well as protocols created with DIVAs pipetting module or Opentrons Protocol Designer.

The user needs to set the basic properties of the project: a name for the project as well as the format of the well plate (24-, 96-, or 384-wells). Additionally, it is possible to select between two algorithms that convert the data to pipetting instructions: The *Simple* algorithm generates instructions from substance to substance and from well to well. While not optimized for speed, this algorithm mimics the conventional workflow. In contrast, the *Advanced* algorithm groups wells with the same volume, thereby speeding up the pipetting process by reducing volume changes of the pipette. If multichannel pipettes are available, the advanced algorithm is able to take them into account for plates with 96-wells or more. Due to the modular design of Pipette Show, it is easy to extend existing or add new algorithms.

On the page required *Substances* can be added, renamed, and reordered. For visual guidance, substance-specific colors are autogenerated but can be user modified as well. The next page *Well Contents* provides an editable spreadsheet where, for each well, the amount of the substances can be modified. Useful tasks like gradient generation or backfill calculations can be applied. Furthermore, the user can directly transfer the protocol to a tablet or perform file actions like loading or saving.

To check the final design, the *Summary* page provides an overview of the just generated experiment and lets the user preview the generated instructions.

**Figure 2:**
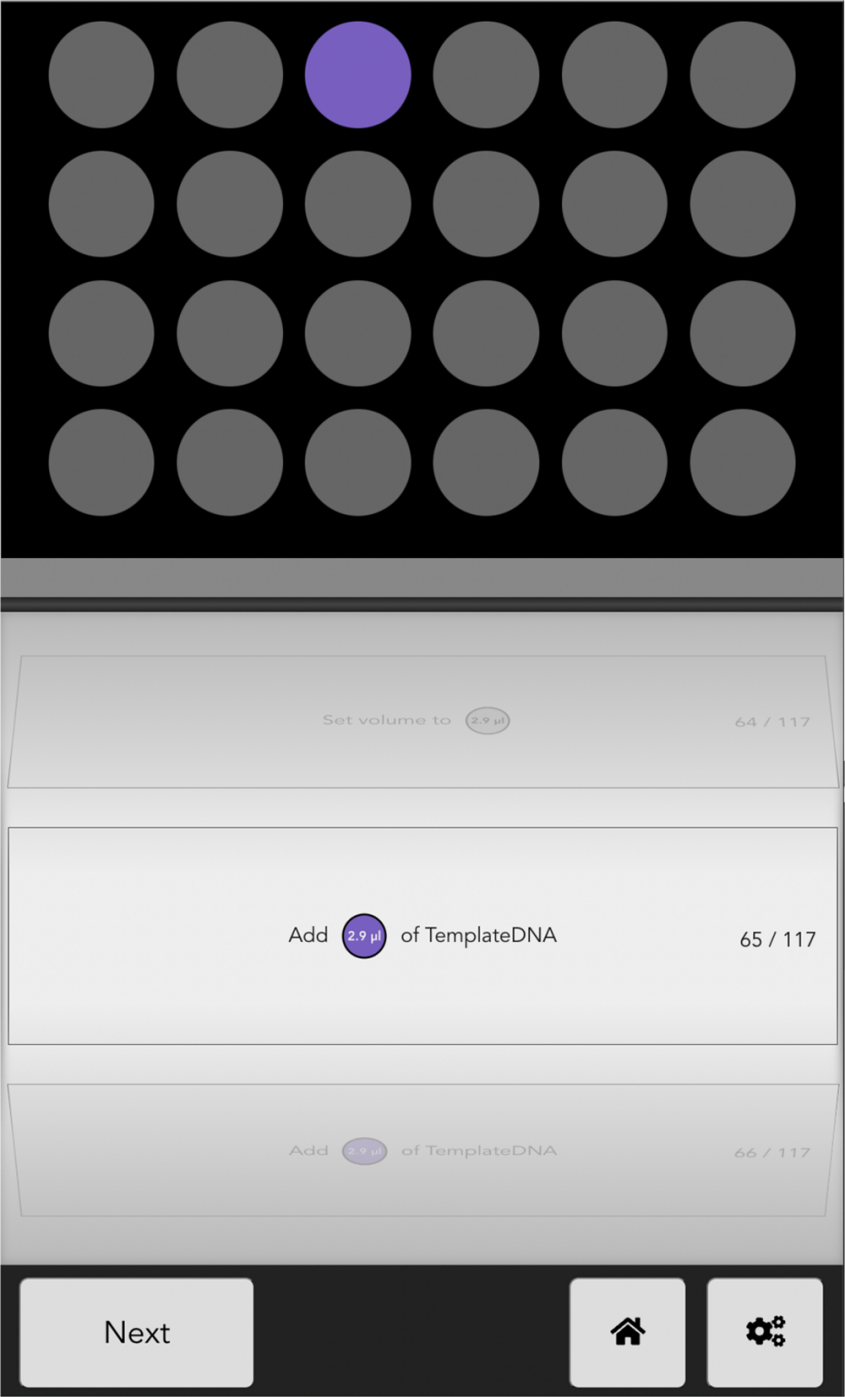
*Show* module without a microplate. At the top, the microplate is loaded after adjusting the size of the plates by dragging the corners. An indicator colored corresponding to the substance shows the target well. The middle section instructs which volumes to pipet of a specific substance and informs the user about substance and volume changes. The lower section is used to advance to the next substance, allows returning to home and modify settings such as going back steps, readjusting the plate size, aborting, or switching between right- and left-hand mode.

Pipette Show provides different ways to export the final experiment to a tablet: If the user wants to download the experiment it can be exported as a .CSV or .PIP file (a .PIP file is a .JSON file that fulfills certain properties that are important for Pipette Show). Alternatively, it is possible to save the files to Google Drive. Pipette Show also provides a more ergonomic way of transferring the experiment via a QR code. This QR code can be scanned with the tablet used to support the pipetting and directly opens the project in the *Show* module (Figure 2).

## 3 *Show* Module

The conventional use case is to run the *Show* module (Figure 2) on a tablet. As a first step, if not already done via a QR code, a project file needs to be loaded from a local file or a file from Google Drive. After that, one mounts the microplate to the tablet and uses the corresponding function to adjust the display to the real size of the microplate. A parametric SCAD file for a possible holder can be found on the Pipette Show web page. This functionality of Pipette Show allows running the *Show* module on various devices with a minimum screen size of 8 IN. Upon starting the protocol, the *Show* module guides through the full pipetting task. Necessary steps are shown on the bottom of the screen and simultaneously the currently active well is highlighted via a back-light. To provide the same usability for right and left-handed users, the *Show* module allows flipping the position of all buttons.

## 4 Future Development

After making an alpha-version of Pipette Show available for user testing, we created a survey to learn which missing features should be implemented. As a result, we added support for 8- and 12-channel pipettes and changed the color of the back-lighting of the wells.

As an open-source project (MIT license), Pipette Show welcomes code contributions from the community. To simplify the addition of further features, the code is structured into modular and extendable libraries that provide distinct functionalities, like, e.g., instruction generation algorithms or data-manipulation tools.

## 5 Implementation and Availability

Pipette Show is mainly developed in Vue.js (Version 3),^11^ a client-side JavaScript framework. The full code is shared on a public git repository https://github.com/Global-Biofoundries-Alliance/pipette-show, where also the development, feature requests, and bug-tracking are organized.

Besides running Pipette Show directly via its Web site https://pipette-show.de, it is also possible to install Pipette Show on a local server, be it for development or to include private cloud solutions.
